# Gut ghrelin regulates hepatic glucose production and insulin signaling via a gut-brain-liver pathway

**DOI:** 10.1186/s12964-019-0321-y

**Published:** 2019-01-25

**Authors:** Yao Lin, Zerong Liang, Liping He, Mengliu Yang, Dongfang Liu, Harvest F. Gu, Hua Liu, Zhiming Zhu, Hongting Zheng, Ling Li, Gangyi Yang

**Affiliations:** 1 Department of Endocrinology, the Second Affiliated Hospital, Chongqing Medical University, Chongqing, 400010 China; 2 The Key Laboratory of Laboratory Medical Diagnostics in the Ministry of Education and Department of Clinical Biochemistry, College of Laboratory Medicine, Chongqing Medical University, Chongqing, 400010 China; 3 Center for Pathophysiology, School of Basic Medicine and Clinical Pharmacy, China Pharmaceutical University, Nanjing, 211198 China; 4 Department of Pediatrics, University of Mississippi Medical Center, 2500 North State Street, Jackson, Mississippi MS 39216-4505 USA; 5 Department of Hypertension and Endocrinology, Daping Hospital, Third Military Medical University, Chongqing Institute of Hypertension, Chongqing, 400010 China; 6 Department of Endocrinology, Xinqiao Hospital, Third Military Medical University, Chongqing, 400037 China

**Keywords:** Insulin resistance, Glucose homeostasis, Duodenum, Ghrelin

## Abstract

**Background:**

Ghrelin modulates many physiological processes. However, the effects of intestinal ghrelin on hepatic glucose production (HGP) are still unclear. The current study was to explore the roles of intestinal ghrelin on glucose homeostasis and insulin signaling in the liver.

**Methods:**

The system of intraduodenal infusion and intracerebral microinfusion into the nucleus of the solitary tract (NTS) in the normal chow-diet rats and pancreatic-euglycemic clamp procedure (PEC) combined with [3-^3^H] glucose as a tracer were used to analyze the effect of intestinal ghrelin. Intraduodenal co-infusion of ghrelin, tetracaine and Activated Protein Kinase (AMPK) activator (AICAR), or pharmacologic and molecular inhibitor of *N*-methyl-D-aspartate receptors within the dorsal vagal complex, or hepatic vagotomy in rats were used to explore the possible mechanism of the effect of intestinal ghrelin on HGP.

**Results:**

Our results demonstrated that gut infusion of ghrelin inhibited duodenal AMP-dependent protein kinase (AMPK) signal pathways, increased HGP and expression of gluconeogenic enzymes, and decreased insulin signaling in the liver of the rat. Intraduodenal co-infusion of ghrelin receptor antagonist [D-Lys^3^]-GHRP-6 and AMPK agonist with ghrelin diminished gut ghrelin-induced increase in HGP and decrease in glucose infusion rate (GIR) and hepatic insulin signaling. The effects of gut ghrelin were also negated by co-infusion with tetracaine, or MK801, an N-methyl-D-aspartate (NMDA) receptor inhibitor, and adenovirus expressing the shRNA of NR1 subunit of NMDA receptors (Ad-*sh*NR1) within the dorsal vagal complex, and hepatic vagotomy in rats. When ghrelin and lipids were co-infused into the duodenum, the roles of gut lipids in increasing the rate of glucose infusion (GIR) and lowering HGP were reversed.

**Conclusions:**

The current study provided evidence that intestinal ghrelin has an effect on HGP and identified a neural glucoregulatory function of gut ghrelin signaling.

**Electronic supplementary material:**

The online version of this article (10.1186/s12964-019-0321-y) contains supplementary material, which is available to authorized users.

## Background

It is well established that nutrients can stimulate the release of gut hormones, such as cholecystokinin and glucagon-like-peptide1, which are involved in the modulation of feeding and gastrointestinal function [1–3]. Recent reports have showen that some hormones or anti-diabetic agents, such as cholecystokinin and metformin, can regulate hepatic glucose production (HGP) in the gut through a neuronal network [4, 5]. Therefore, it is important to further investigate the physiological role of novel signaling molecules within the duodenum in the modulation of glucose metabolism through an intestine-brain-liver pathway.

Ghrelin is a 28-amino acid peptide originally identified in human and rat stomachs as an endogenous natural ligand of growth hormone secretagogue receptor 1a (GHS-R1a). It is produced in X/A-like cells of oxyntic mucosa [6]. Subsequently, ghrelin is found in other parts of the gut and in other tissues, such as the kidney and hypothalamus [7]. As a multifaceted gut-brain peptide, it stimulates growth hormone secretion and regulates a variety of physiological processes such as stimulating food intake and fat deposition resulting in weight gain and adiposity in adult animals [8] and humans [9]. In addition, it has been reported that ghrelin promotes insulin secretion, and decreases glucose-stimulated insulin secretion in animals and humans [10, 11]. Importantly, circulating ghrelin levels are found to change under energy balance conditions. For instance, the levels are elevated with anorexia nervosa, cachexia, or fasting, and reduced after food intake and in obese subjects [12–15]. Therefore, ghrelin may have a crucial role in the development of insulin resistance (IR)-related diseases. Accumulating evidence has indicated that ghrelin is involved in glucose metabolism in peripheral tissues and the central nervous system. In the gastrointestinal tract, two types of ghrelin cells have been found; i.e. closed-type cells and opened-type cells [16], and it is well known that an open endocrine cell can release its hormone into the lumen [17]. Importantly, a previous study demonstrated that ghrelin infusion into the duodenal lumen stimulates pancreatic enzyme secretion in rats [18]. However, the impact of gut ghrelin on HGP and insulin signaling remains unknown. In the current study, we have investigated the roles of gut ghrelin to modulate HGP via a neuronal network.

## Methods

### Animal preparation

Nine-week-old male Sprague-Dawley rats (300-350 g) were fed in individual cages and allowed ad libitum access to food and water. Animals were given 7 days to adapt before the experiments. Rats underwent duodenal cannulation as previously described [19] and infusion catheters were placed in the proximal duodenum 1.5–2 cm downstream of the pyloric sphincter. Several additional groups of rats were stereotaxically implanted with a bilateral steel guide cannula positioned 2 mm above the caudomedial nucleus of the solitary tract (NTS). Bilateral catheters were inserted into the dorsal vagal complex (DVC) targeting NTS for injection. Accurate cannula placement was histologically confirmed by consumption of more than 1.5 g of chow within 60 min following a parenchymal injection of 24 μg of 5-thio-D-glucose (Sigma) in 0.1 μl of artificial cerebrospinal fluid (aCSF) per side and adenovirus expressing green fluorescent (Ad-GFP) (Additional file 1: Figure S1) [20]. Indwelling catheters were placed into the internal jugular vein and carotid artery for infusion and blood sampling during the pancreatic-euglycemic clamp (PEC).

### Selective hepatic branch vagotomy

**Fig. 1:**
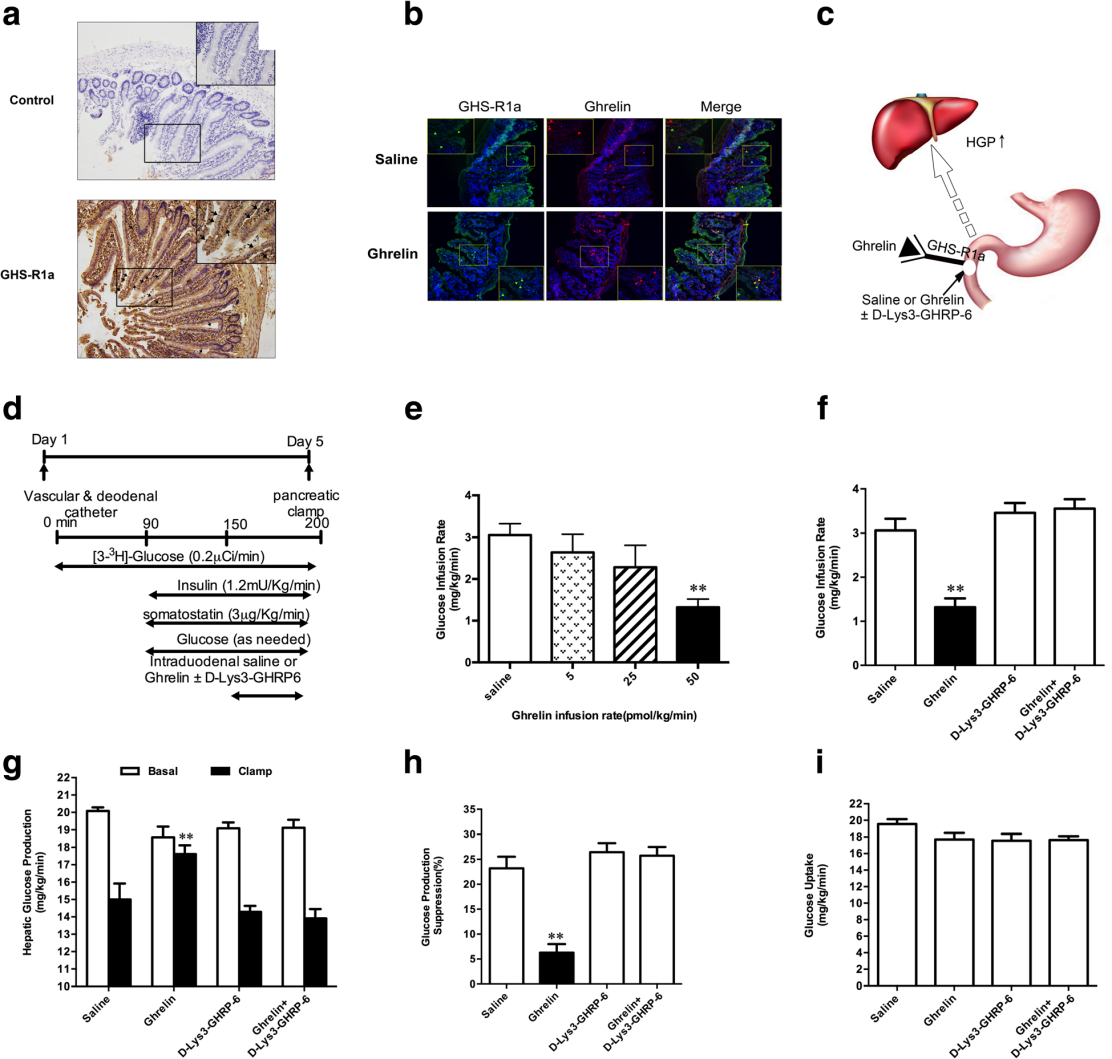
Gut ghrelin increases liver glucose production through ghrelin-receptor (GHS-R1a). **a** Photomicrographs of GHS-R1a immunostaining incubated with saline or GHS-R1a antibody in rat duodenal tissues. Arrowheads indicate positive cells for GHS-R1a staining. **b** Immunofluorescent images of duodenal tissue sections stained for GHS-R1a (green) and ghrelin (red). **c** Schematic representation of working hypothesis. Ghrelin with or without GHS-R1a antagonist ([D-Lys^3^]-GHRP-6) was infused through a duodenal catheter. **d** Experimental procedure and clamp protocol. Duodenal catheter or venous and arterial catheters were implanted on day 1. The pancreatic clamp studies were performed on day 5. **e** Gut ghrelin decreased GIR in dose dependent manner. **f** Cumulative GIR during the steady-state of clamp. **g** HGP. **h** Suppression of HGP during the clamp period expressed as the percentage reduction from basal HGP. **i** Glucose uptake. GIR, the rate of glucose infusion; HGP, hepatic glucose production. Data are means ± SEM, ^**^ *P* < 0.01 vs. all other groups. (*n* = 7 for saline or ghrelin treated group; *n* = 5 per group for all other groups)

Seven days prior to PEC, hepatic branch vagotomy (HVAG) or sham operation (SHAM) was performed in the separate cohorts of rats as previously described [21]. Meanwhile, the rats selected for duodenum infusion and clamp study were implanted with additional catheters in the proximal duodenum, the right internal jugular vein and left carotid artery (Fig. 1d).

### Adenovirus infection in DVC

In the post-stereotaxic surgery, two subgroups of rats were immediately injected with 1 μl adenovirus expressing the *sh*RNA of NR1 subunit of NMDA receptors (Ad-*sh*NR1) or mismatch sequence (Ad-MM) to the NR1 subunit of the NMDA receptor as previously described [22, 23]. Six days after adenoviral injection, vascular catheterization was performed as described above. The sequences of Ad-*sh*NR1 and the mismatch were listed as follows: 5’-GAATGTCCATCTACTCTGATTCA AGAGATCAGAGTAGATGGACATTC-3′ or the mismatch (MM) 5’-TTCTCCG AACGTGTCACGTAA-3′. A viral titer in plaque forming unit (pfu)/ml was determined by endpoint dilution assay (adenovirus containing small hairpin RNA- NR1: 1.0 × 10 ^11^ pfu/ml; MM: 1.0 × 10 ^11^ pfu/ml).

### Pancreatic-euglycemic clamp procedure (PECs)

PECs were performed with awake and unrestrained rats. A continuous infusion of HPLC–purified [3-H^3^] glucose (PerkinElmer, MA, USA; 6 μCi bolus, 0.2 μCi/min) was initiated at 0 min and maintained throughout the clamp. The PECs were started at time 90 min after the tracer infusion to allow the tracer to reach a steady state and continued until the end of the PECs. Somatostatin (3 μg/kg/min) was infused intravenously together with insulin (1.2 mU/kg/min) to inhibit endogenous insulin secretion. A 25% glucose solution was started at time 90 min and periodically adjusted every 5–10 min to maintain blood glucose at ~ 6 mmol [24]. Intraduodenal infusions were initiated from t = 150 to 200 min to examine the impacts of different treatments on glucose metabolism. In a separate cohort of rats undergoing NTS treatment procedures, MK-801, an N-methyl-D-aspartate (NMDA) receptor inhibitor (0.03 ng/min, Sigma-Aldrich, St Louis, MO, USA), was given at t = 90 to 200 min until the end of the PECs. Blood was collected by the jugular vein at 0, 60, 90, 180, 190 and 200 min for measurement of ghrelin and other parameters. At the completion of the PEC, tissue samples were collected and stored at − 80 °C for further analysis.

### Intraduodenal infusion protocols

To determine the dose-dependent effects of intraduodenal ghrelin on the glucose infusion rate (GIR) during PECs, different concentrations of ghrelin (0, 5, 25, 50 pmol/kg/min, Enzo Alexis, Inc. Lausen, Switzerland, #ALX-157-022-MC05) were continuously infused to the duodenum through the duodenal catheter. The lowest GIR was observed at a ghrelin infusion rate of 50 pmol/kg/min. For all experiments, this concentration was infused into the duodenum. In addition, the following materials were also infused into the duodenum (0.6 ml/h) during the PECs when required: (1) saline; (2) ghrelin receptor antagonist ([D-Lys^3^]-GHRP-6, 1500 pmol/kg/min, Sigma-Aldrich Inc., St. Louis, MO, USA; #G4535); (3) tetracaine (0.01 mg/min, Sigma-Aldrich USA); (4) AMP-dependent protein kinase (AMPK) agonist (AICAR, Sigma, St. Louis, MO, USA; #A9978); (5) 20% Intralipids (0.03 kcal/min, Sino-Swed Pharmaceutical Corp. Ltd).

### Fasting-feeding experiment

After the duodenal and vascular surgery, rats were allowed 4 days to recover. Two subgroups of rats were fasted beginning at 5 PM on the day before the experiment. Rats were fed for 20 min after fasting and then subjected to an intraduodenal infusion of either saline or ghrelin (50 pmol/kg/min) for 30 min (from − 10~20 min, Fig. 3g). Blood glucose levels were measured at t = 0, 10, and 20 min. Cumulative food intake was measured at the end of this study.

### Immunohistochemistry and immunofluorescence for ghrelin and its receptor expression in duodenum

Duodenum sections were pretreated in 0.5% Triton (Sigma Aldrich) and 1.5% BSA (Sigma Aldrich) in PBS for 15 min at 25 °C. Successively, the sections were incubated with GHS-R1a polyclonal antibody. The immunoreaction was revealed by using the horseradish peroxidase-labeled sheep anti-rabbit antibody. For immunofluorescence analysis, frozen sections of intestinal tissue were incubated with primary antibodies at 4 °C overnight and then washed with PBS. The sections were incubated with secondary antibody and then counterstained with 4′, 6- diamidino- 2-phenylindole (DAPI) for nuclear detection [25]. Primary antibodies included anti-GHS-R1a (Abcam, Cambridge, UK, ab85104), anti-ghrelin (Santa Cruz Biotechnology, Dallas, TX, USA, sc-293422).

### Analysis of gene expression at mRNA and protein levels

Real-time quantitative PCR was performed as described previously [26]. Forward and reverse primer pairs were: 5’-CACCTTGACACTACACCCTT-3′ and 5’-GTGGCT GTGAACACCTCT-3′ for glucose- 6- phosphatase (G6Pase); 5’-AGTCA CCATCAC TTCCTGGAAGA-3′ and 5’-GGTGCAGAATCGCGAGTT-3′ for phosphoenolpyruvate carboxykinase (PEPCK); 5′-AAGATGCCTCCTGTGACT-3′ and 5′-GATGACCGAAGTGCTTGT-3′ for proliferator-activated receptor γ co- activator 1α (PGC-1α); 5’-CCCTGAACCCTAAGGCCAACCGTGAAAA-3′ and 5’-TCTCCGGAGTCCATCACAATGCCTGTG- 3′ for β-actin. Protein analysis was performed with Western Blots as described previously [24]. Primary antibodies included anti-insulin receptor (InsR) (#3025)/ phospho-InsR (Tyr1150/1151, #3024), anti- AMP-dependent protein kinase (AMPK) (#2532)/ phosphor- AMPK (Thr172, #2531), and anti-AKT kinase (AKT) (#9272)/phospho-AKT (Ser473, #4060) (Cell Signaling Technology, Beverly, MA, USA); anti- PEPCK; and β-actin (Santa Cruz Biotechnology, Dallas, TX, USA, sc-47778).

### Analytical procedures of glucose, glucagon, insulin, c-peptide and ghrelin concentrations

Blood glucose levels were examined with the glucose oxidase method. Plasma glucagon, c-peptide, and ghrelin concentrations were measured using a commercial ELISA Kit (CUSABIO Inc. Wuhan, China; #CSB-E12800r/#CSB-E05067r /#CSB -E13167r). Plasma [3-H^3^] glucose–specific activity was measured as described [27].

### Statistical analyses

All results are expressed as means ± SEM. Analysis of variance with a least significant difference post hoc test was used to compare the mean values between multiple groups and the two-sample and unpaired Student’s *t*-test was used for two group comparisons. *P* < 0.05 was considered as the significance.

## Results

### Duodenal ghrelin increases hepatic glucose production

To examine whether GHS-R1a was expressed in the duodenum, GHS-R1a immunoreactivity was performed in the duodenum tissue of rat. GHS-R1a was detected in Brunner’s gland epithelium and lamina propria with a predominantly basolateral membrane-associated staining (Fig. 1a). We performed immunofluorescence using GHS-R1a and ghrelin antibodies, and found that intraduodenal ghrelin infusion could markedly increase ghrelin expression and the co-localization of ghrelin and GHS-R1a in the intestinal wall relative to gut saline infusion, further indicating that ghrelin might interact with GHS-R1a in intestinal mucosal cells (Fig. 1b; Additional file 1: Figure S2).

To investigate whether gut ghrelin can regulate HGP (Fig. 1c), we activated gut ghrelin signaling via direct intraduodenal ghrelin infusion in vivo. Experimental procedure and clamp protocol were described in Fig. 1d. First, we infused ghrelin (0, 5, 25, 50 pmol/kg/min) into the duodenum of rat and the GIR demonstrated a dose-dependent decrease during PEC (Fig. 1e). The 50 pmol/kg/min infusion of ghrelin decreased GIR by approximately 2.3 fold, and thus it was selected for following experiments. Plasma insulin, glucose and FFA levels were kept at approximately basal values during the PEC (Additional file 1: Table S1). Importantly, the GIR required to maintain euglycemia was significantly decreased by the administration of gut ghrelin, suggesting a decrease of insulin sensitivity (Fig. 1f). In addition, intraduodenal ghrelin significantly increased HGP when compared with intraduodenal saline (Fig. 1g and h) independent of changes in glucose uptake (Fig. 1i) during the PEC. However, gut ghrelin infusion did not increase in ghrelin levels in the circulation and portal vein (Additional file 1: Table S2 and Table S3). Thus, the increase of ghrelin level in the duodenum decreased the GIR and increased the HGP without changes in circulating ghrelin or insulin levels, suggesting that ghrelin infusion in the duodenum has local (mucosa) but not systemic effects.

### Inhibition of GHS-R1a prevents the effects of duodenal ghrelin on HGP

To further investigate whether GHS-R1a in intestinal mucosal cells is required for the role of gut ghrelin on HGP, we infused [D-Lys^3^]-GHRP-6, a GHSR1a antagonist, with ghrelin into the gut. The co-infusion of [D-Lys^3^]-GHRP-6 and ghrelin in the gut fully eliminated the roles of gut ghrelin to decrease GIR (Fig. 1f) and increase HGP (Fig. 1g and h), while glucose uptake was unchanged (Fig. 1i). The infusion of [D-Lys^3^]-GHRP-6 alone did not influence GIR and HGP (Fig. 1f-h). Therefore, GHS-R1a in intestinal mucosal cells is necessary for gut ghrelin to increases HGP.

### The role of duodenal ghrelin on glucose kinetics through a neuronal network

**Fig. 2:**
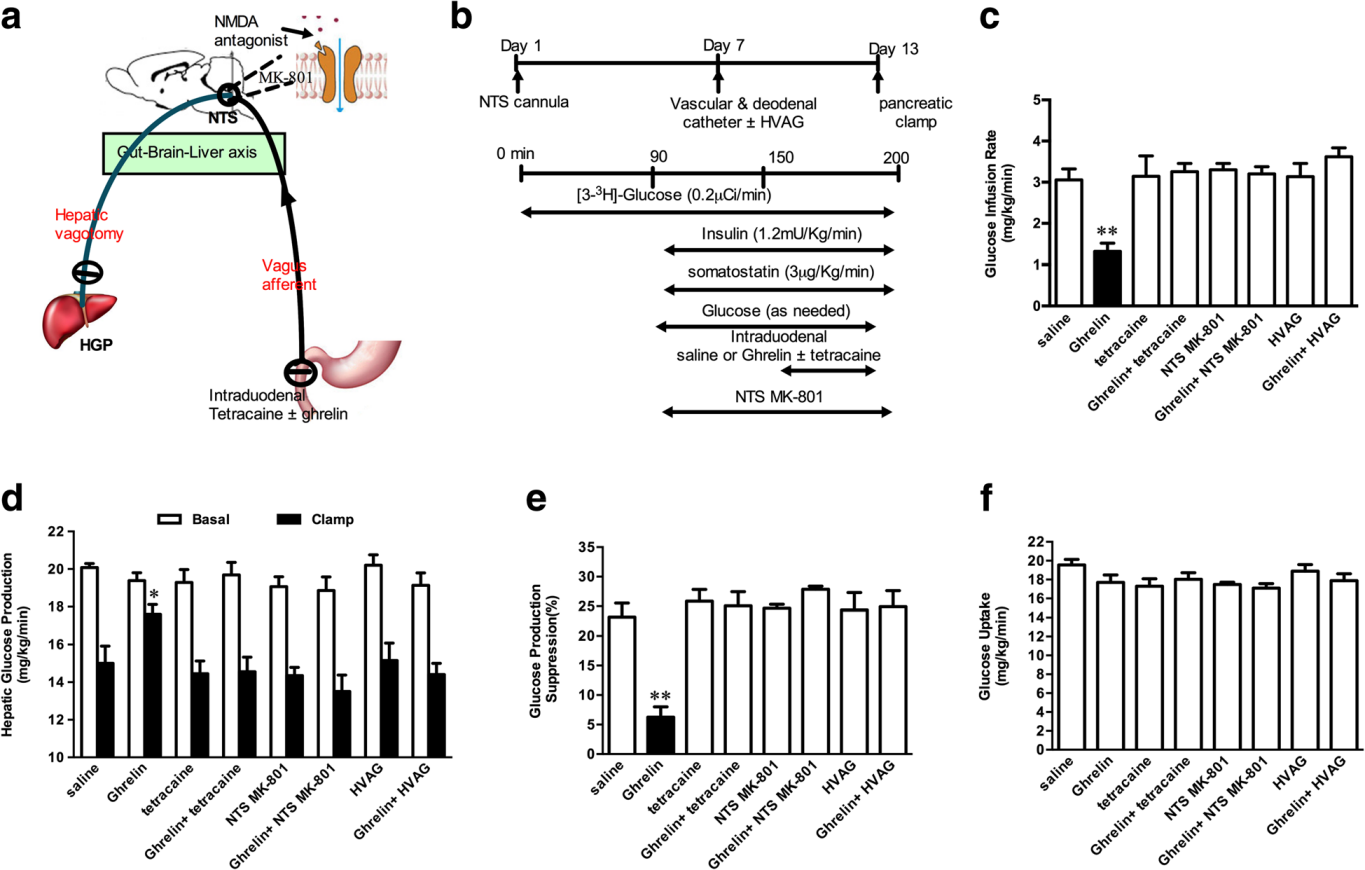
Gut ghrelin increases hepatic glucose production by activating a gut-brain-liver neurocircuitry. **a** Schematic representation of the working hypothesis. Gut ghrelin was coinfused with tetracaine, which abolishes the ascending neuronal signal to the brain. A subgroup of rats was given MK-801, an NMDA receptor inhibitor, directly into the NTS. In another study, gut ghrelin was infused into rats with HVAG. **b** Experimental procedure and clamp protocol. Stereotaxic surgeries were conducted on day 1. A duodenal catheter or venous and arterial catheters were implanted on day 7. HVAG was performed immediately before the implantation of the duodenal and vascular catheters. **c** and **d** Gut ghrelin infusion decreased GIR and increased HGP. Rats that received tetracaine in the gut, MK-801 in the NTS or HVAG failed to respond to duodenal ghrelin to decrease the GIR and increase HGP. **e** Suppression of HGP during the clamp expressed as the percentage decrease from basal HGP. **f** Glucose uptake was unchanged in all groups. NTS, the nucleus of the solitary tract; HVAG, hepatic vagotomy; NMDA, *N*-methyl- D-aspartate; GIR, glucose infusion rate; HGP, hepatic glucose production. Values are shown as mean ± SEM. *P < 0.05, **P < 0.01, vs. all other groups. (*n* = 7 for saline or ghrelin treated group; *n* = 5 per group for all other groups)

To investigate how gut ghrelin signal is transferred to the liver to increase HGP, we used three independent approaches to evaluate whether a neuronal network involving the effects of gut ghrelin on HGP (Fig. 2a). We co-infused both the ghrelin and the tetracaine (a blocker of ryanodine receptor) into the gut (Fig. 2b). The infusion of tetracaine alone did not change GIR (Fig. 2c) and HGP (Fig. 2d and e). Importantly, the ability of intraduodenal ghrelin to decrease GIR (Fig. 2c) and increase HGP was fully eliminated by the co-infusion of ghrelin and tetracaine (Fig. 2c-e), suggesting that gut vagal afferent fibers are required for the effect of ghrelin signaling on HGP. The co-infusion of ghrelin and tetracaine had no effect in glucose uptake (Fig. 2f).

Vagal afferent nerves terminate at the NTS within the DVC. NMDA receptors in the caudomedial nucleus of the NTS mediate intraduodenal signals initiated by hormone or nutrients to regulate HGP. To examine whether gut ghrelin signals were also mediated by the NMDA receptors, we directly infused MK-801 into the NTS to inhibit glutamatergic neurotransmission during the intraduodenal infusion of ghrelin and PEC (Fig. 2a and b). We found that the administration of MK-801 alone did not alter GIR (Fig. 2c) and HGP (Fig. 2d and e). However, when the intraduodenal infusion of ghrelin was concomitantly administered with NTS MK-801, the ability of gut ghrelin to decrease the GIR (Fig. 2c) and increase the HGP (Fig. 2d and e) was fully attenuated. Glucose uptake rate was no changed by NTS MK-801 (Fig. 2f).

To further evaluate whether gut ghrelin activates NR1 subunit to regulate HGP, we used molecular approaches to inhibit NR1. The injection of Ad-*sh*NR1 into the DVC significantly decreased NR1 protein levels (Additional file 1: Figure S3). When gut ghrelin was concomitantly administered with DVC Ad-*sh*NR1, the abilities of gut ghrelin to decrease the GIR and increase HGP were attenuated (Additional file 1: Figure S4). Finally, we infused ghrelin into the duodenum of rats that underwent HVAG to block the neuro-communication from brain to liver to investigate the descending pathway that NTS NMDA receptors relay the signal generated by gut ghrelin (Fig. 2a). Compared with the SHAM group, HVAG did not lead to the changes of circulating glucagon and C-peptide levels (Additional file 1: Table S4). HVAG alone did not affect the change of glucose kinetics. However, HVAG fully negated the ability of duodenal ghrelin to decrease the GIR (Fig. 2c) and increase HGP (Fig. 2d and e). Taken together, these results indicate that gut ghrelin signaling was relayed by the gut vagal afferent fibers to NTS, hepatic vagal efferent nerve, and then liver to increase HGP.

### Duodenal ghrelin blockades the role of lipid infusion on glucose turnover

**Fig. 3:**
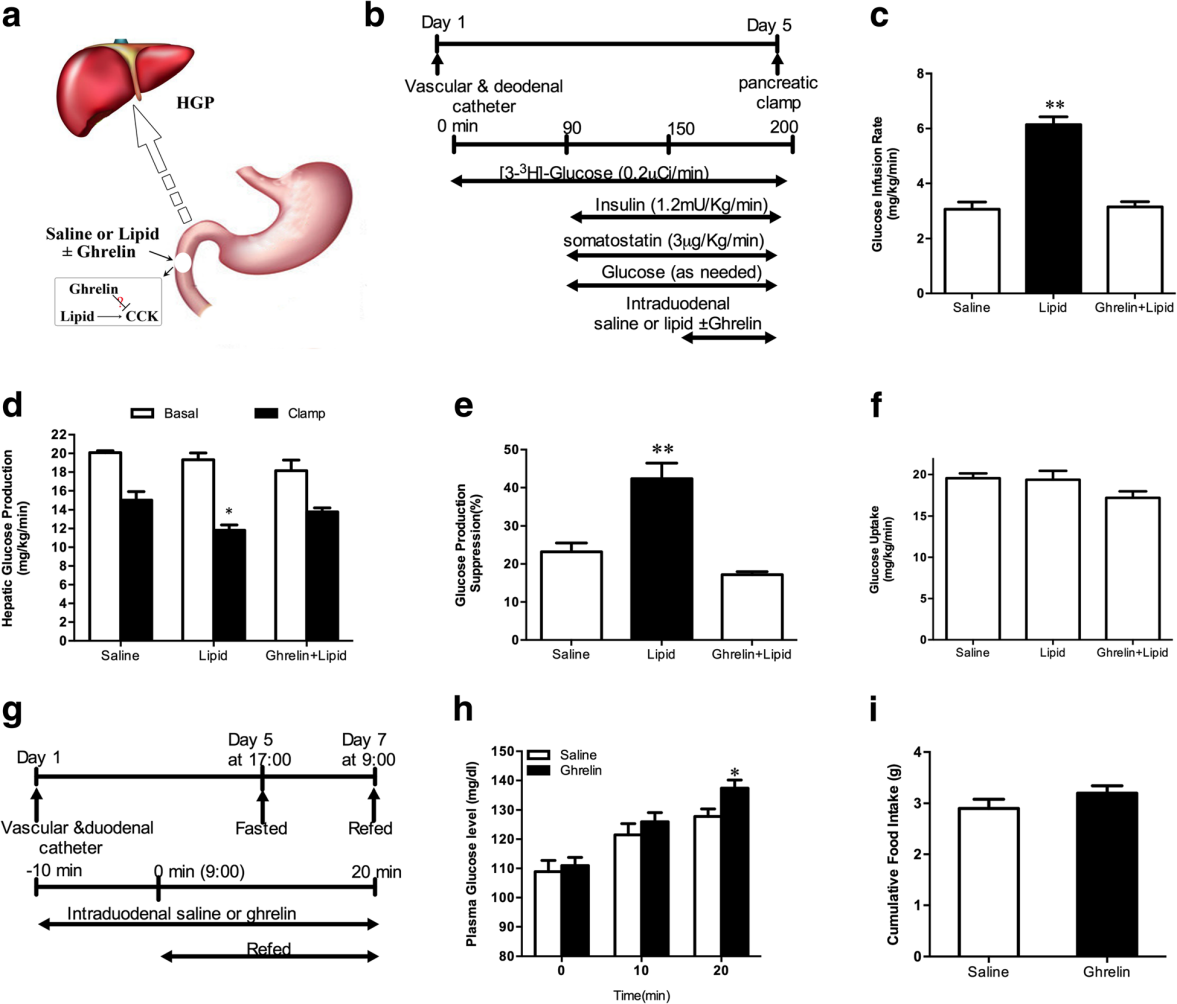
Gut ghrelin infusion disrupts the gut nutrient sensing-related mechanisms. **a** Schematic representation of the working hypothesis. Lipid with or without ghrelin, or saline was infused through a duodenal catheter. **b** Experimental procedure and clamp protocol. **c-e** Gut lipids infusion increased the GIR (**c**), and decreased HGP (**d** and **e**). When duodenal lipid was co-infused with ghrelin, the effects of lipids on GIR and HGP were abolished. **f** Glucose uptake was unchanged in all groups. **g** - **i** The effect of gut ghrelin on glucose homeostasis during fasting-refeeding. **g** Schematic representation of the experimental design. A duodenal catheter, the internal jugular vein and carotid artery catheters were implanted on day 1. Rats were subjected to a 40 h fasting from 5 p.m. on day 5 until 9 a.m. on day 7. Ten minutes before the completion of the forty-hour fast, rats were infused with intraduodenal saline or ghrelin (n = 5 per group). Rats were refed on regular chow at time 0 min where food intake and blood glucose were monitored for 20 min. **h** Plasma glucose levels during refeeding. **i** Cumulative food intake during refeeding. GIR, glucose infusion rate; HGP, hepatic glucose production. Values are shown as mean ± SEM. *P < 0.05, **P < 0.01 vs. saline or other groups

To assess the role of ghrelin in gut nutrition-sensing mechanisms, we infused lipid with and without ghrelin into the duodenum during PEC (Fig. 3a and b). As expected, lipid infusion into the gut of rats increased GIR (Fig. 3c) and lowered HGP (Fig. 3d and e). When ghrelin and lipid were co-infused into the duodenum, the roles of gut lipid to increase GIR and to lower HGP were reversed (Fig. 3c-e), whereas glucose uptake was unchanged (Fig. 3f). These data suggest that gut ghrelin infusion may block the gut lipid-sensing mechanisms.

### Duodenal ghrelin alters glucose homeostasis during fasting-refeeding

We further investigated the effects of gut ghrelin on nutrient sensing-related mechanisms induced by a physiological fasting-refeeding protocol (Fig. 3g). When the rats were fasted and then re-fed, blood glucose rises rapidly, but this elevation was limited due to a decrease of hepatic gluconeogenesis or an increase of glycogen synthesis [28]. We deduce if gut ghrelin can disrupt nutrient sensing related mechanisms activated by refeeding to limit HGP and lower blood glucose, blood glucose should be further elevated. Five days after vascular and duodenal catheterizing (Fig. 3g), rats fasted for 40 h. Blood glucose was elevated at 10–20 min of refeeding in gut saline infused rats (Fig. 3h). However, gut ghrelin further increased blood glucose at 20 min of refeeding compare with gut saline (Fig. 3h). Food intake was similar in both groups (Fig. 3i). These results suggest that gut ghrelin disrupts glucose homeostasis during fasting-refeeding.

### Gut ghrelin regulates glucose metabolism through the inhibition of AMPK signal in intestinal mucosal cells

**Fig. 4:**
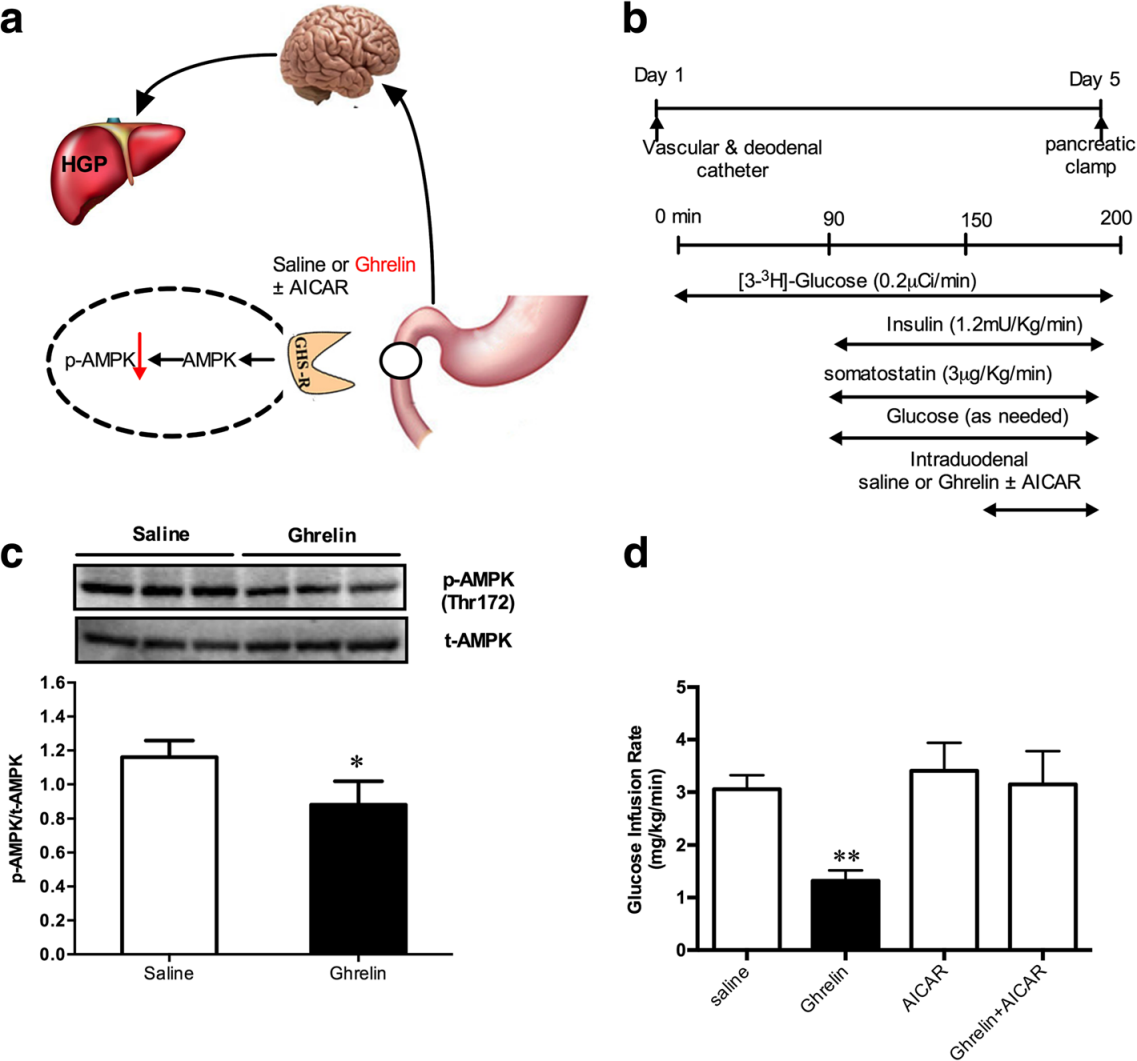
Gut ghrelin increases HGP through AMPK inhibition. **a** Schematic of the working hypothesis. **b** Experimental procedure and clamp protocol. Ghrelin was infused with or without AICAR, an AMPK activator, through a duodenal catheter. **c** Representative western blots of AMPK in the mucosal layer of the duodenum. **d** Gut ghrelin infusion decreased the GIR. When duodenal ghrelin was co-infused with AICAR, the effects of ghrelin on GIR were abolished. GIR, glucose infusion rate. Values are shown as mean ± SEM. *P < 0.05, **P < 0.01 vs. other groups. (*n* = 7 per group)

The effects of ghrelin on AMPK activity in peripheral tissues and the hypothalamus have been demonstrated [29]. AMPK is also expressed in the intestine [30]. Therefore, we further explored the effect of gut ghrelin on AMPK activation (Fig. 4a). Surgical procedures and pancreatic clamp are shown in Fig. 4b. We first infused ghrelin into the gut and examined the phosphorylation of AMPK (Thr172) in duodenal mucosa layer of rats. We discovered that the levels of AMPK (Thr172) phosphorylation were significantly decreased in the duodenal mucosa by ghrelin infusion (Fig. 4c), suggesting a link between ghrelin signaling and AMPK pathway. We then activated duodenal mucosal AMPK signaling via intraduodenal co-infusion of ghrelin with the AMPK activator, AICAR (0.5. 2.5 and 5 nmol/kg/min). We found that AICAR infusion at 5 nmol/ kg/ min increased GIR significantly (Additional file 1: Figure S5). In order to avoid the interference of AICAR, 5 nmol/kg/min was selected for the following experiments. The treatment of gut ghrelin plus AICAR fully abolished the role of ghrelin to decrease GIR (Fig. 4d).

### Duodenal ghrelin increases expression of PEPCK, G6Pase and PGC-1α in liver

**Fig. 5:**
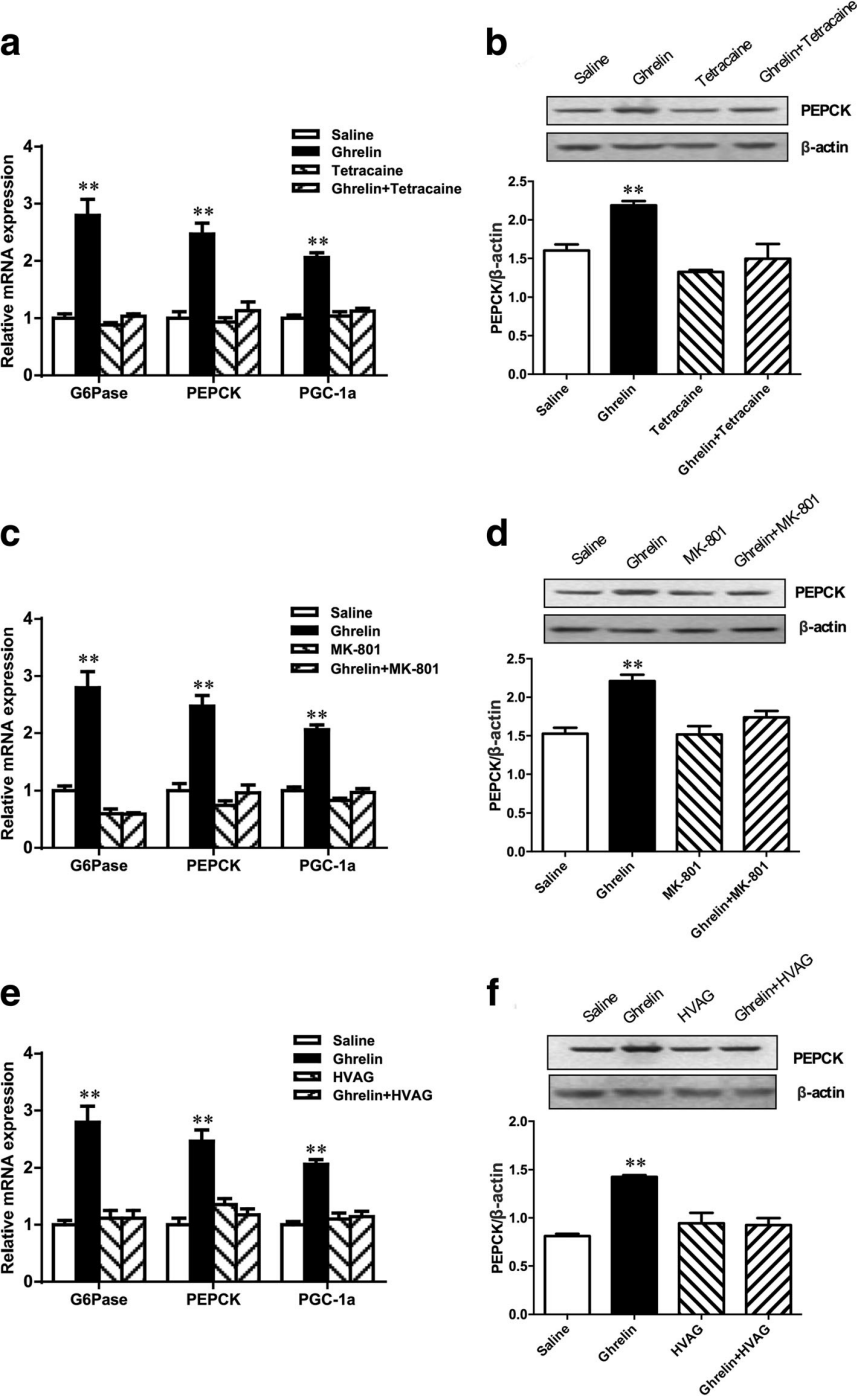
Duodenal ghrelin increases hepatic PEPCK, G6Pase and PGC-1α expression through a gut-brain-liver neurocircuitry. Intraduodenal ghrelin infusion in rats increased hepatic PEPCK, G6Pase and PGC-1α mRNA expression (**a**) and PEPCK protein expression (**b**). Rats that received tetracaine (**a** and **b**), MK-801 in the NTS (**c** and **d**) or HVAG (**e** and **f**) failed to respond to duodenal ghrelin to increase hepatic PEPCK, G6Pase and PGC-1α expression. NTS, nucleus of the solitary tract; HVAG, hepatic vagotomy. Values are shown as mean ± SEM. ** *P* < 0.01 vs. other groups

Because gut ghrelin increased the HGP, we then investigated whether gut ghrelin led to changes in phosphoenolpyruvate carboxykinase (PEPCK) and glucose-6- phosphatase (G6Pase) expressions, two key gluconeogenic enzymes. As expected, gut ghrelin significantly increased insulin-stimulated mRNA expression of PEPCK and G6Pase in the liver compared with gut saline infusion (Fig. 5a). The protein expression of PEPCK in the liver also significantly increased in gut ghrelin infused rats (Fig. 5b). Furthermore, gut ghrelin infusion led to an increase of peroxisome proliferator-activated receptor gamma coactivator1-alpha (PGC-1α) mRNA expression (Fig. 5a). These data indicated that gut ghrelin attenuated the inhibitory effects of insulin on hepatic PEPCK, G6Pase, and PGC-1α, and thus led to increased HGP. Parallel to the PEC studies, the intraduodenal infusion of combining tetracaine with ghrelin negated the ability of gut ghrelin to increase hepatic expression of PEPCK, G6Pase and PGC-1α mRNA and PEPCK protein (Fig. 5 a and b). Similarly, the infusion of intraduodenal ghrelin plus NTS MK-801 also prevented the ghrelin-induced an increase in PEPCK, G6Pase and PGC-1α mRNA expressions and PEPCK protein levels in the liver of rats (Fig. 5 c and d). When MK-801 alone was infused to NTS, hepatic expressions of PEPCK and G6Pase were comparable with the gut saline infusion (Fig. 5 c and d). Furthermore, intraduodenal ghrelin infusion in HVAG rats did not alter the expression of PEPCK, G6Pase and PGC-1α mRNA or PEPCK protein in the liver (Fig. 5 e and f). Therefore, denervation of the hepatic branch of the vagus nerve was sufficient to abolish the increased expression of PEPCK, G6Pase, and PGC-1α induced by gut ghrelin.

### Duodenal ghrelin inhibits insulin signal in liver

**Fig. 6:**
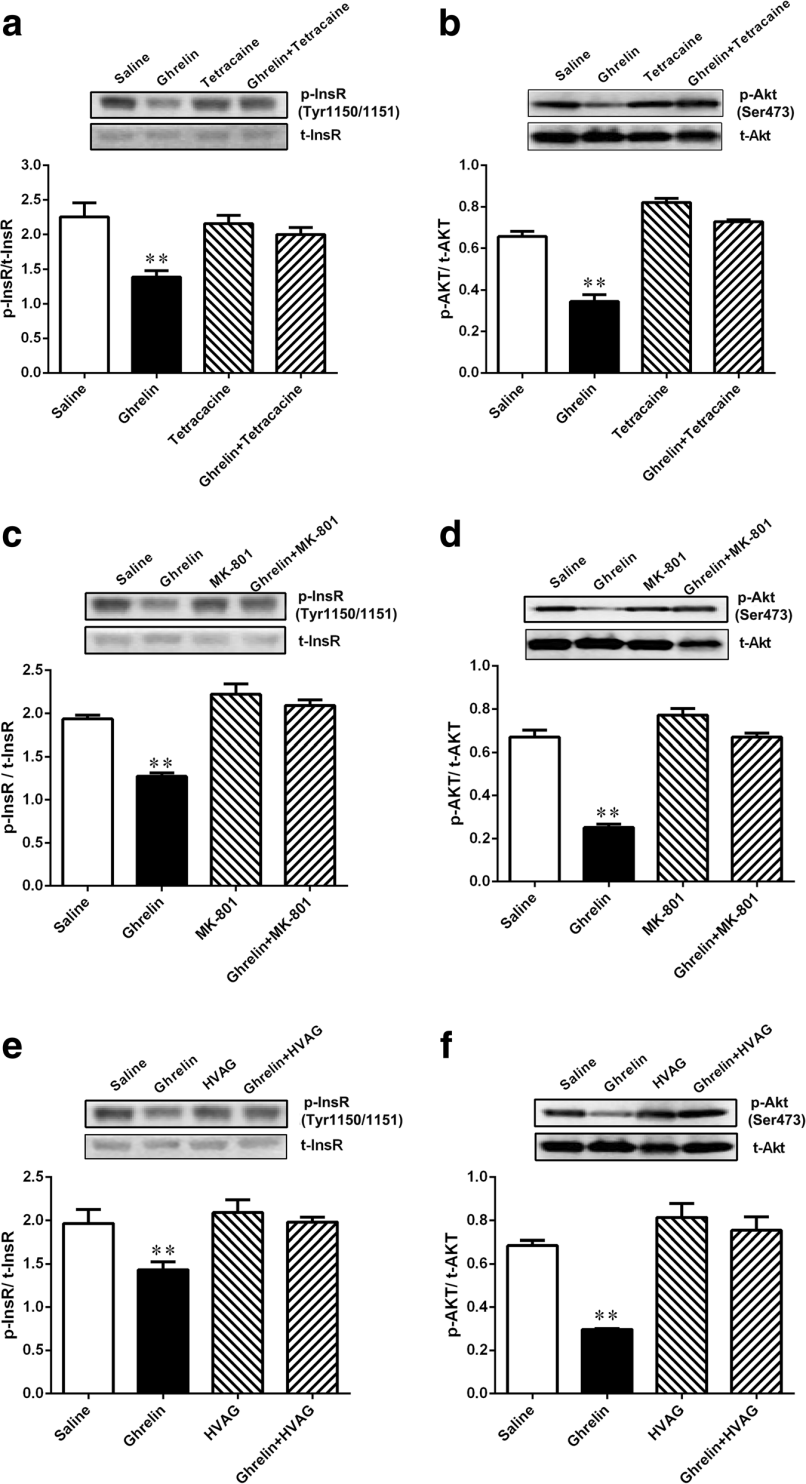
Gut ghrelin attenuated hepatic insulin signaling through a gut-brain- liver neurocircuitry. Intraduodenal ghrelin infusion decreased the phosphorylation of InsR (**a**) and AKT (**b**) in the liver of rats. Rats that received tetracaine (**a** and **b**), MK-801 in the NTS (**c** and **d**) or HVAG (**e** and **f**) failed to respond to duodenal ghrelin to decrease the phosphorylation of InsR (Tyr 1150/1151) and AKT (Ser473) in the liver of rats. InsR, insulin receptor; AKT, protein kinase B; NTS, nucleus of the solitary tract; HVAG, hepatic vagotomy. Values are shown as mean ± SEM. ** *P* < 0.01 vs. other groups

To explore the effects of gut ghrelin on insulin signaling, we measured the phosphorylation of proteins in the insulin signaling pathway in the liver by Western blots. The results showed that duodenal ghrelin resulted in a significant reduction in the phosphorylation of InsR (Tyr 1150/1151) and AKT (Ser473) in the liver of rats (Fig. 6 a and b). However, when tetracaine and ghrelin were concomitantly infused into the gut, the effect of ghrelin on phosphorylation of InsR (Tyr 1150/1151) and AKT (Ser473) was abolished (Fig. 6 a and b).

To further understand whether NTS NMDA receptors mediate the gut ghrelin-induced inhibition of hepatic insulin signals, we infused MK-801 into the NTS. The infusion of intraduodenal ghrelin along with the NTS infusion of MK801 fully negated gut ghrelin-induced decreases in the phosphorylation of InsR (Tyr1150/1151) and AKT (Ser473) in the liver (Fig. 6 c and d). In addition, to evaluate whether gut ghrelin signal was relayed to the liver to inhibit insulin signaling cascade via hepatic vagal efferent nerve. We thus repeated duodenal ghrelin infusion tests in HVAG rats. HVAG abolished gut ghrelin-induced inhibition of the phosphorylation of InsR (Tyr 1150/1151) and AKT (Ser473) in the liver (Fig. 6 e and f).

## Discussion

In diabetes and obesity, HGP elevation is a significant feature of the IR state [31]. Therefore, it is important to assess whether different gut-peptide hormones, such as glucagon-like-peptide1 (via intraduodenal infusion or oral ingestion) regulate HGP. In the current study, we investigated that gut ghrelin infusion increased HGP without changes in peripheral circulating ghrelin and insulin levels. Data demonstrated that 1) Ghrelin activated GHS-R1a in the gut to trigger a gut-brain-liver neuronal network resulting in an increased HGP; 2) Gut ghrelin negated the glucoregulatory function of gut nutrient sensing in a fasting/refeeding experiment; 3) Gut ghrelin infusion blocked the gut lipid-sensing mechanisms; 4) Duodenal ghrelin infusion increased the expressions of key gluconeogenic genes and inhibited insulin signal in the liver; 5) Gut ghrelin infusion mediated glucose metabolism through the inhibition of AMPK pathway in intestinal mucosal cells.

Given that dietary lipids and glucose stimulate local releases of gut-peptide hormones from the mucosal cells [26], it is important to investigate whether different gut-peptide hormones regulate HGP in the preabsorptive states. Here, we activated duodenal ghrelin signaling via direct intraduodenal infusion. Our data show that the intraduodenal ghrelin infusion increased HGP and hepatic expressions of G6Pase and PEPCK, and inhibited hepatic insulin signaling. In addition, PGC-1α mRNA expression in the liver was significantly increased in the ghrelin infusion rats which may directly enhance gluconeogenesis through the transcriptional regulation of gluconeogenic enzymes [32]. These changes were independent of alterations in circulating ghrelin and insulin levels. Thereby, these outcomes may be mediated by a mechanism that normally occurs in a local compartment, i.e. paracrine signaling in the gut. In previous human and animal studies, elevated circulating ghrelin levels in the periphery have a similar effect on glucose metabolism and insulin action [33, 34]. Herein, we have demonstrated that the increase of ghrelin in the gut triggers a gut hormone sensor to promote gluconeogenesis and increase HGP via activating gluconeogenic enzymes and inhibiting hepatic insulin action and that the control of HGP by gut ghrelin requires the presence of duodenal ghrelin receptors. Hereby, we further confirmed that ghrelin plays key roles in communicating glucose homeostasis.

Accumulating evidence suggests that the neuronal network of intestine-brain and brain-liver modulates energy balance [35–38]. Furthermore, the existence of an intestine-brain-liver neuronal network has been recently found in glucoregulatory function [4, 26]. Several studies have investigated the effects of intestinal nutrients and hormones on HGP and fat metabolism [26, 39, 40]. In the current study, we assessed the role of ghrelin in gut nutrition-sensing mechanisms and found that gut lipid infusion increased GIR and lowered HGP during PEC, consistently with the previous report [39]. The inhibition of HGP fully accounted for the increase in GIR. There could be several explanations for HGP inhibition induced by gut lipid: 1) the formation of LCFA-CoAs; 2) Intestinal hormones released by gut lipid infusion [2, 39, 41]; 3) activating an intestine–brain–liver neural axis to inhibit HGP [39]. However, the roles of gut lipid in increasing GIR and lowering HGP were reversed by gut ghrelin. Thus, this implicated that gut ghrelin infusion might block the gut lipid-sensing mechanisms. Furthermore, we have dissected the physiological role of gut ghrelin in regulating glucose homeostasis and insulin action via a gut-brain-liver axis. Because peripheral administration of ghrelin has been shown to increase food intake via the vagal afferents [42], we first examined if the vagal afferent system is the major pathway conveying gut ghrelin signals for feeding and the regulation of energy homeostasis to the brain. We infused topical anesthetic tetracaine locally to inhibit the neurotransmission of vagal afferent fibers innervating the intestinal mucosa. Tetracaine administration significantly negated the duodenal ghrelin-induced increase in HGP and attenuated hepatic insulin signals, indicating that gut ghrelin may regulate HGP through vagal afferent innervation for neurotransmission. In fact, it has been shown that interruption of the vagal afferent pathway prevents ingestion induced by ghrelin and attenuates ghrelin-induced growth hormone secretion [43].

It has been reported that the caudal hindbrain NTS senses afferent neuronal signals to maintain energy balance [2, 3]. NMDA receptors in the gut-recipient neurons (glutamatergic transmitter) in NTS of the hindbrain mediate gut signals initiated by hormones or nutrients to regulate glucose homeostasis [3, 44]. We, therefore, tested whether glutamatergic transmission in the NTS relays afferent neuronal signals from the duodenal ghrelin to the liver to increase HGP. When NMDA receptor was inhibited by NMDA receptor blocker MK-801 and Ad-*sh*NR1 the effects of gut ghrelin on HGP and hepatic insulin signals were fully blocked. These results support that NTS transmission relays afferent neuronal signals triggered by gut ghrelin to increase HGP and attenuate hepatic insulin signals.

A previous study has demonstrated that both hepatic vagal afferent and efferent pathways are important in feeding and regulating energy balance [21]. Dissection of hepatic vagal branch removes neural transmission not only from the liver to brain but also reversely from the brain to liver, while leptin and glucagon-like-peptide1 directly control liver and/or white adipose tissue metabolism, and most of those signals require an intact sympathetic nervous system [45, 46]. Therefore, we hypothesized that vagal pathway from the brain to liver mediates the gut ghrelin-induced changes in hepatic insulin signals and HGP. Consistently with this hypothesis, hepatic vagotomy prevented the ability of intestine ghrelin to decrease GIR and raise HGP. Thus, the NTS relays a signal generated by intestinal ghrelin to the liver through hepatic vagal efferent pathways. These results collectively indicate that duodenal ghrelin triggers an intestine-brain-liver neuronal network to increase hepatic IR and thus increases HGP.

AMPK is an important regulatory enzyme of cholesterol and TG syntheses, fatty acid oxidation, glycogen synthesis, and glucose output in lipogenesis and lipolysis of liver and adipose tissue [47]. Previous study has shown that ghrelin activates AMPK both in vivo and in vitro [48]. Importantly, the recent studies have suggested that duodenal AMPK is required for some compounds, including metformin and resveratrol, to lower HGP [5, 49]. In this study, we found that the duodenal mucosa of gut ghrelin-treated rats had a lower ratio of phosphorylated AMPK (Thr172) to total AMPK than that of saline-treated animals, suggesting lower AMPK activity. We then intraduodenally co-infused the AMPK activator AICAR and ghrelin, and discovered that AICAR fully abolished the ability of gut ghrelin to decrease GIR. The results indicate that duodenal AMPK inhibition is required for the metabolic effects of gut ghrelin. Furthermore, we demonstrate that duodenal ghrelin can block the gut lipid-sensing mechanisms and disrupt glucose homeostasis during refeeding through a neuronal network. In the previous study, ghrelin has been shown to block the action of cholecystokinin through inhibiting cholecystokinin-stimulated nuclear translocation of early growth response factor-1 and vagal afferent nerve discharge [50]. Therefore, the effects of ghrelin on gut nutrient sensing mechanisms may be due to the inhibiting of cholecystokinin action.

**Fig. 7:**
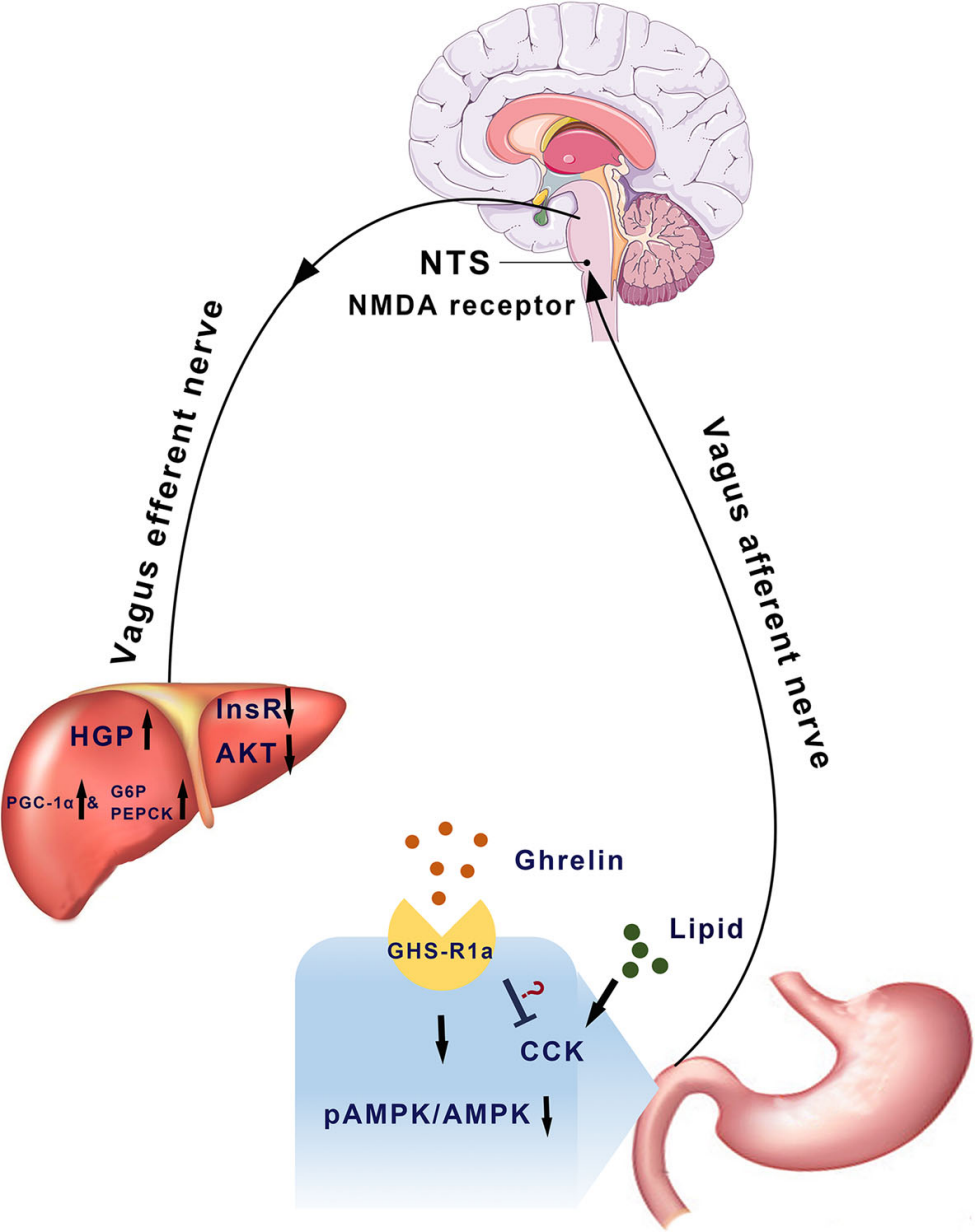
A schematic model showing the effects of duodenal ghrelin on hepatic glucose fluxes and insulin signals. Ghrelin binds to its receptor, GHS-R1a, on the duodenum and inhibits mucosal AMPK. This signal is delivered to the NTS by vagus afferent nerve and activates the neurons in hindbrain region and NMDA receptors. Finally, the signal is relayed from the NTS to the liver via the efferent branch of the vagal nerve to increase HGP and inhibit insulin signal

It appears plausible that upper intestine nutrients, such as lipids, stimulate or inhibit the release of gut-peptide hormones, such as ghrelin, from mucosal cells, which bind to their receptors on vagal afferents nerves in the duodenum and then activate mucosal AMPK. By activating vagal afferent nerves, hormone derived signals arrive at the NTS, bind to the NMDA receptors, and activate the neurons in the hindbrain region. Finally, these signals are relayed from the NTS to the liver via the efferent branch of the hepatic vagal nerve to regulate HGP (Fig. 7).

## Conclusions

The current study provides the first evidence that ghrelin inhibits a previously unappreciated duodenal AMPK-dependent neuronal pathway to increase HGP and deteriorates hepatic IR in rats. The data also elucidate the existence of a hormone- induced gut-brain-liver neuronal network in the regulation of glucose homeostasis and insulin signals in vivo*.*

## Additional file

Additional file 1: **FigureS1.** Photograph of a representative histologic section demonstrating a cannula tip center in the left medial subnucleus of NTS (above), Original magnification, × 40. Photograph of the brainstem. **Figure S2.** Comparison of GHS-R1a and ghrelin immunoreactivity in duodenal mucosa in gut ghrelin and control rats. **Table S1.** Biochemical parameters under basal and clamped conditions. **Table S2.** Circulating ghrelin levels at baseline and steady-state in different groups. **Table S3.** Circulating and portal vein ghrelin concentrations. **Figure S3.** Molecular knockdown of NR1 subunit of the NMDA receptor. **Figure S4.** Molecular knockdown of NTS NR1 negates the effect of gut ghrelin on GIR and HGP. **Table S4.** Glucagon and c-peptide levels in SHAM and HAVG group during PEC. **Figure S5.** Gut AICAR increased GIR in gut ghrelin-infused rat. (DOCX 9482 kb)

## Abbreviations

AKT: protein kinase B
AMPK: AMP-dependent protein kinase
DVC: the dorsal vagal complex
G6Pase: glucose- 6- phosphatase
GHS-R1a: growth hormone secretagogue receptor 1a
GIR: glucose infusion rate
HGP: hepatic glucose production
HVAG: hepatic branch vagotomy
InsR: insulin receptor
IR: insulin resistance
MM: mismatch sequence
NMDA: N-methyl-D-aspartate
NR1: N-methyl-D-aspartate receptor 1
NTS: nucleus of the solitary tract
PEC: pancreatic-euglycemic clamp
PEPCK: phosphoenolpyruvate carboxykinase
PGC-1α: proliferator-activated receptor γ co-activator 1α
SHAM: sham operation
TG: Triglyceride
