# Supplementary material for: Gut ghrelin regulates hepatic glucose production and insulin signaling via a gut-brain-liver pathway
Source: Cell Commun Signal. 2019 Jan 25;17:8. doi: 10.1186/s12964-019-0321-y (PMC6347823; doi:10.1186/s12964-019-0321-y)
Supplement: Supplementary file 1 — FigureS1. Photograph of a representative histologic section demonstrating a cannula tip center in the left medial subnucleus of NTS (above), Original magnification, × 40. Photograph of the brainstem. Figure S2. Comparison of GHS-R1a and ghrelin immunoreactivity in duodenal mucosa in gut ghrelin and control rats. Table S1. Biochemical parameters under basal and clamped conditions. Table S2. Circulating ghrelin levels at baseline and steady-state in different groups. Table S3. Circulating and portal vein ghrelin concentrations. Figure S3. Molecular knockdown of NR1 subunit of the NMDA receptor. Figure S4. Molecular knockdown of NTS NR1 negates the effect of gut ghrelin on GIR and HGP. Table S4. Glucagon and c-peptide levels in SHAM and HAVG group during PEC. Figure S5. Gut AICAR increased GIR in gut ghrelin-infused rat. (DOCX 9482 kb) [file 12964_2019_321_MOESM1_ESM.docx]

**Additional file 1**

**Figure S1**


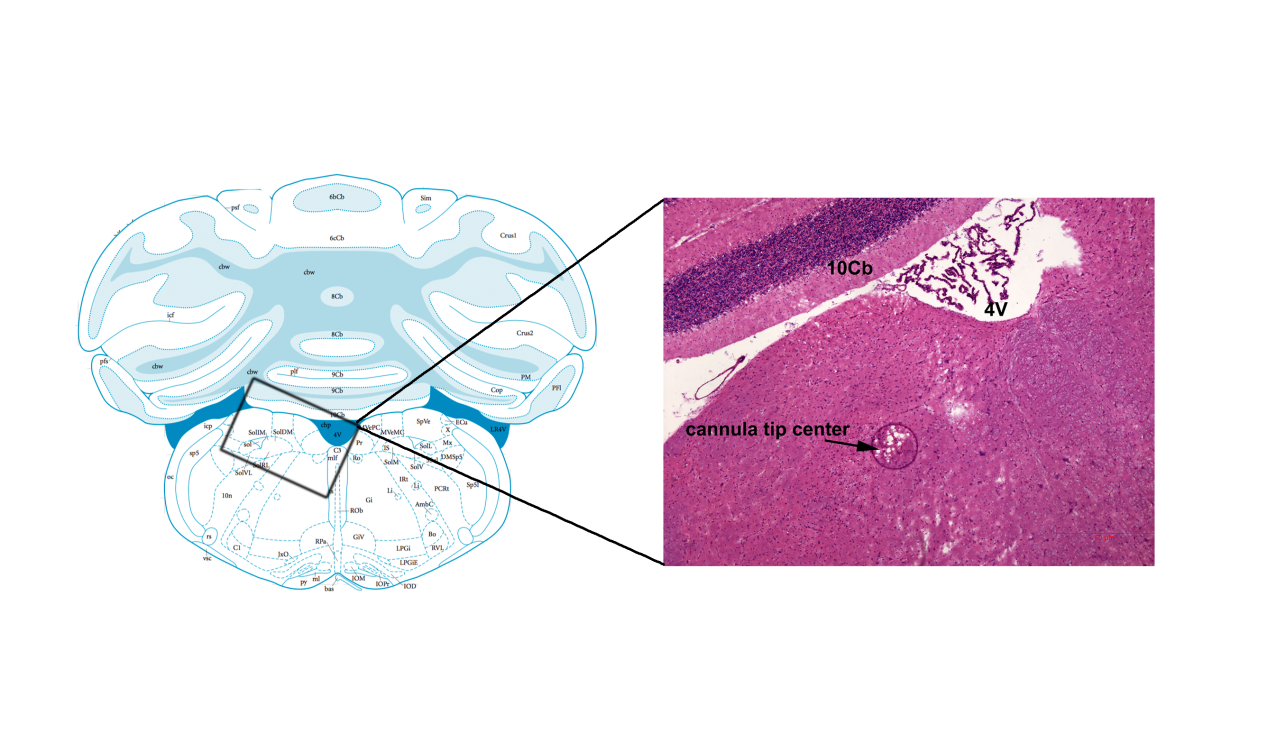

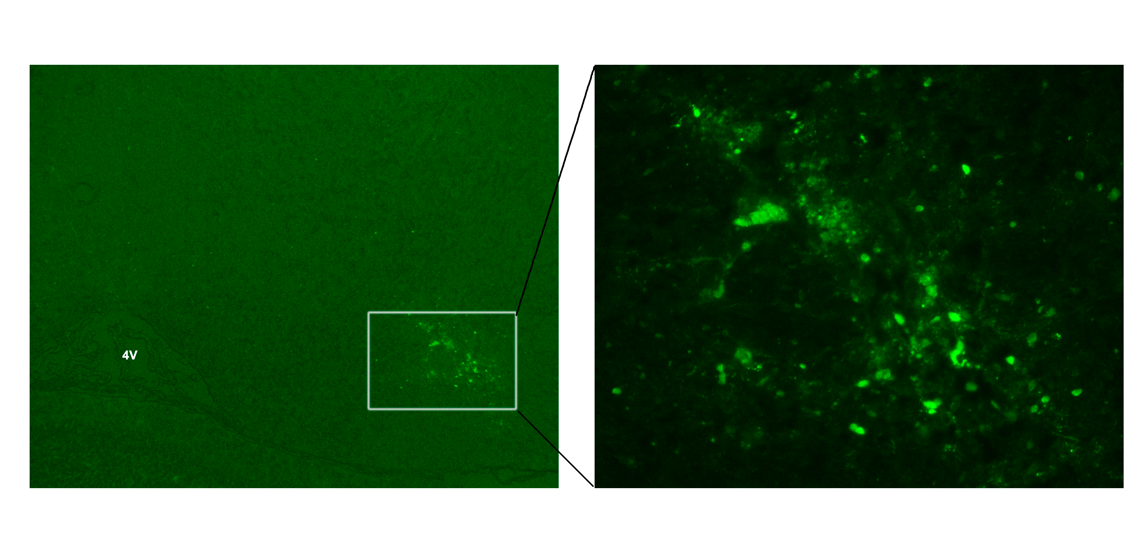


**Figure S1** Photograph of a representative histologic section demonstrating a cannula tip center in the left medial subnucleus of NTS (above), Original magnification, ×40. Photograph of the brainstem. Rat was microinjected with adenovirus expressing green fluorescent (Ad-GFP) into NTS to show the location of microinjection site (blew), Original magnification, ×40 or ×200. NTS, nucleus of the solitary tract; 10Cb, 10th cerebellar lobule; 4V, fourth ventricle.

**Figure S2**


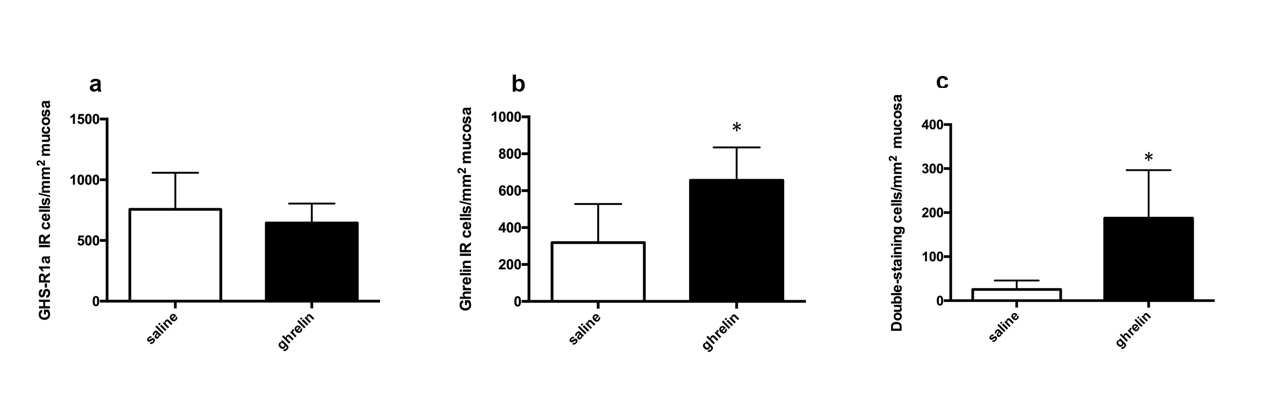


**Figure S2** Comparison of GHS-R1a and ghrelin immunoreactivity in duodenal mucosa in gut ghrelin and control rats. Number of GHS-R1a immunoreactive cells (A), ghrelin immunoreactive cells (B) and GHS-R1a-ghrelin double-staining cells (C) per mm^2^ in mucosa of duodenum. IR, immunoreactive; Saline, gut saline infusion; Ghrelin, gut ghrelin infusion. Values are shown as mean ± SEM, **P* < 0.05.

**Table S1**

| Treatment | | Insulin (ng/ml) | Glucose  (mmol/l) | TG  (mmol/l) | TC  (mmol/l) | FFA  (mmol/L) |
| --- | --- | --- | --- | --- | --- | --- |
| **Saline** | | | |  |  |  |
| Basal | 0.83 ± 0.14 | | 7.0 ± 0.4 | 0.71 ± 0.09 | 1.79 ± 0.15 | 1.07 ± 0.17 |
| Clamp | 0.80 ± 0.12 | | 7.5 ± 0.3 | 0.68 ± 0.08 | 1.66 ± 0.14 | 1.03 ± 0.21 |
| **Ghrelin** | | | |  |  |  |
| Clamp | 0.82 ± 0.10 | | 7.0± 0.4 | 0.71 ± 0.11 | 1.69 ± 0.14 | 0.91 ± 0.09 |
| **D-Lys3-GHRP6** | | |  |  |  |  |
| Clamp | 0.84±0.09 | | 6.6± 0.3 | 0.7 ± 0.1 | 1.65 ± 0.12 | 0.99 ± 0.12 |
| **Ghrelin+D-Lys3-GHRP6** | | | | | | |
| Clamp | 0.80±0.11 | | 7.2± 0.3 | 0.67 ± 0.10 | 1.82 ± 0.24 | 0.99 ± 0.08 |
| **Tetracaine** | | | |  |  |  |
| Clamp | 0.89 ± 0.19 | | 6.2 ± 0.2 | 0.70 ± 0.06 | 1.65 ± 0.16 | 1.02 ± 0.18 |
| **Ghrelin+Tetracaine** | | | |  |  |  |
| Clamp | 0.97 ± 0.22 | | 7.9 ± 0.3 | 0.61± 0.09 | 1.72 ± 0.14 | 0.98 ± 0.26 |
| **NTS MK-801** | | | |  |  |  |
| Clamp | 0.82 ± 0.31 | | 7.6 ± 0.4 | 0.58 ± 0.18 | 1.57 ± 0.14 | 0.82 ± 0.28 |
| **Ghrelin+ NTS MK-801** | | | |  |  |  |
| Clamp | 0.85 ± 0.11 | | 7.3 ± 0.6 | 0.63 ± 0.10 | 0.87 ± 0.11 | 0.82 ± 0.05 |
| **HVAG** | | | |  |  |  |
| Clamp | 0.94 ± 0.14 | | 7.6 ± 0.5 | 0.56 ± 0.07 | 1.65 ± 0.20 | 1.17 ± 0.05 |
| **Ghrelin+HVAG** | | | |  |  |  |
| Clamp | 1.13 ± 0.10 | | 6.6 ± 0.2 | 0.51 ± 0.18 | 1.75 ± 0.08 | 0.95 ± 0.19 |
| **Lipid** | | | |  |  |  |
| Clamp | 0.93 ± 0.10 | | 7.6 ± 0.3 | 0.51 ± 0.10 | 1.87 ± 0.08 | 1.13 ± 0.19 |
| **Ghrelin+Lipid** | | | |  |  |  |
| Clamp | 0.73± 0.20 | | 7.2 ± 0.5 | 0.58 ± 0.15 | 1.79 ± 0.29 | 1.06 ± 0.22 |

**Table S1** Biochemical parameters under basal and clamped conditions. HVAG, hepatic branch vagotomy; NTS, the nucleus of the solitary tract; TG, Triglyceride; TC, Total cholesterol; FFA, free fatty acid; NTS, Nucleus of the solitary tract. Data are means ± SEM.

**Table S2**

| Groups | Circulating ghrelin (ng/L) | |
| --- | --- | --- |
|  | Baseline Steady-state | |
| Saline | 239.4 ± 12.9 | 223.2 ± 9.7 |
| Ghrelin | 232.8 ± 23.8 | 233.8 ± 18.5 |
| D-Lys3-GHRP6 | 223.1 ± 13.3 | 235.9 ± 10.6 |
| Ghrelin+D-Lys3-GHRP6 | 241.9 ± 13.9 | 223.7 ± 19.0 |
| Tetracaine | 232.5 ± 12.9 | 229.2 ± 7.9 |
| Ghrelin+ tetracaine | 229.2 ± 12.8 | 209.1 ± 8.1 |
| NTS MK-801 | 241.6 ±10.2 | 245.4 ± 6.4 |
| Ghrelin+ NTS MK-801 | 259.9 ±14.2 | 272.3 ± 28.4 |
| HVAG | 268.5 ± 11.0 | 267.3 ± 12.5 |
| Ghrelin+ HVAG | 252.2 ± 24.4 | 269.5 ± 24.3 |
| Lipid | 245.0 ±12.6 | 248.5 ±9.1 |
| Ghrelin+Lipid | 264.5 ±14.5 | 265.3 ± 14.1 |

**Table S2** Circulating ghrelin levels at baseline and steady-state in different groups. HVAG, hepatic branch vagotomy; NTS, the nucleus of the solitary tract; Data are means ± SEM.

**Table S3**

| Infusion | Saline | Ghrelin (ng/L) |
| --- | --- | --- |
| Circulation | 223.2 ± 9.7 | 233.8 ± 18.5 |
| Portal vein | 242.0 ± 9.3 | 254.4 ± 11.2 |

**Table S3** Circulating and portal vein ghrelin concentrations. Data are means ± SEM.

**Figure S3**


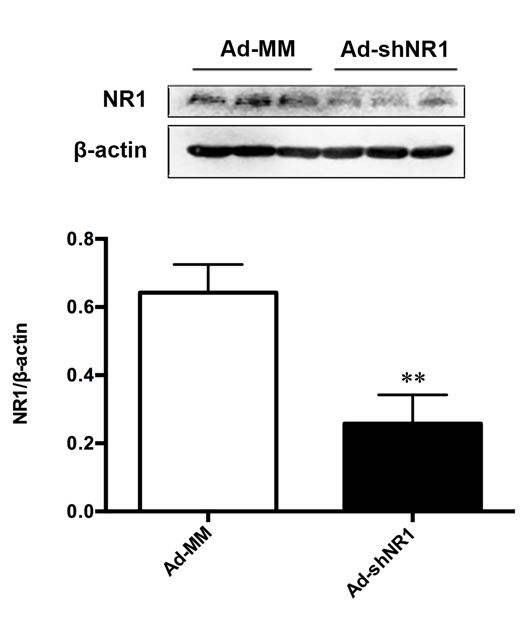


**Figure S3** Molecular knockdown of NR1 subunit of the NMDA receptor. NTS stereotaxic surgery was performed on SD rats. The rats were injected with 3μl of mismatch (MM) or Ad-*sh*NR1 per side of cannulae. NR1, *N*-methyl- D-aspartate receptor1. Values are means ± SEM. ***P* <0.01 *vs.* MM.

**Figure S4**


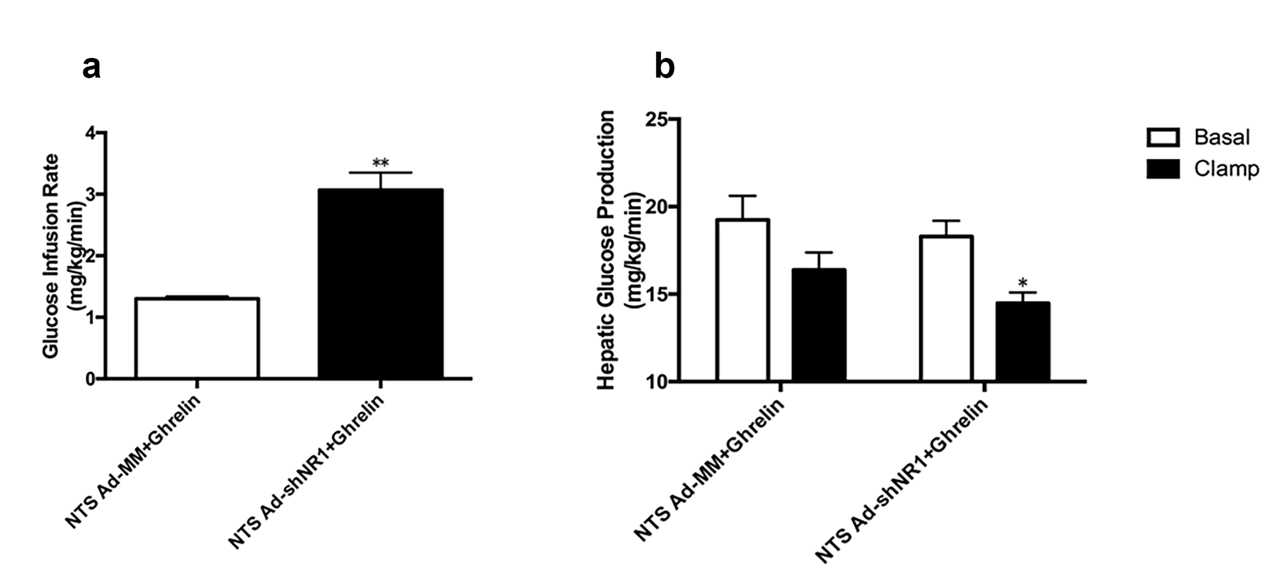


**Figure S4** Molecular knockdown of NTS NR1 negates the effect of gut ghrelin on GIR and HGP. The infusion of gut ghrelin in rats injected with NTS Ad-*sh*NR1 failed to decrease GIR (A) and increase HGP (B). NTS, solitary tract; NR1, *N*-methyl- D-aspartate receptor1; GIR, glucose infusion rate; HGP, hepatic glucose production. Data are shown as mean ± SEM, *vs.* Ad-MM * *P*<0.05 or ***P* < 0.01.

**Table S4**

|  | Glucagon | C-peptide |
| --- | --- | --- |
|  | (ng/L) | (µg/L) |
| **SHAM** |  |  |
| Basal | 50.93±4.05 | 4.16±0.39 |
| Clamp | 32.29±5.69 | 2.39±0.28 |
| **HVAG** |  |  |
| Basal | 51.04±2.64 | 3.99±0.40 |
| Clamp | 31.35±6.08 | 2.13±0..23 |

**Table S4** Glucagon and c-peptide levels in SHAM and HAVG group during PEC. Data are means ± SEM.

**Figure S5**


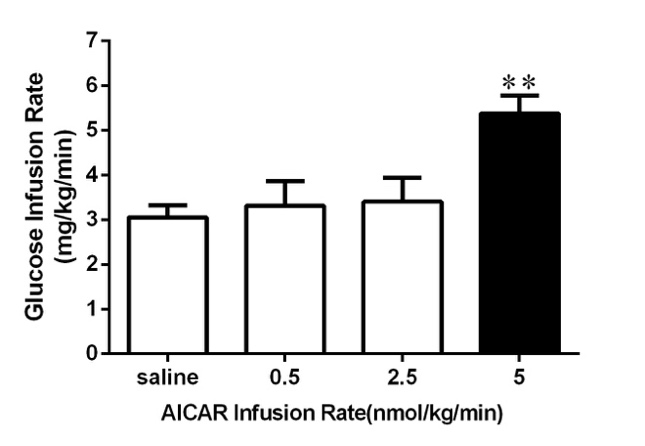


**Figure S5** Gut AICAR increased GIR in gut ghrelin-infused rat. AICAR (0.5. 2.5 and 5 nmol/kg/min) was infused into the duodenum in gut ghrelin- infused rat and the GIR demonstrated an increase during PECs**.** Data are shown as mean ± SEM, ***P*< 0.01 *vs*. saline group.
